# Limited efficacy of West Nile virus vaccines in large falcons (*Falco* spp.)

**DOI:** 10.1186/1297-9716-45-41

**Published:** 2014-04-07

**Authors:** Joke Angenvoort, Dominik Fischer, Christine Fast, Ute Ziegler, Martin Eiden, Jorge Garcia de la Fuente, Michael Lierz, Martin H Groschup

**Affiliations:** 1 Friedrich-Loeffler-Institut, Federal Research Institute for Animal Health, Institute of Novel and Emerging Infectious Diseases, Südufer 10, 17493 Greifswald, Insel Riems, Germany; 2 Clinic for Birds, Reptiles, Amphibians and Fish, Justus Liebig University Giessen, Frankfurter Str. 91-93, 35392 Giessen, Germany; 3 Roc Falcon S.L., Finca Caballera Alta, 25283 Odèn, Lleida, Spain

## Abstract

West Nile virus (WNV) can lead to fatal diseases in raptor species. Unfortunately, there is no vaccine which has been designed specifically for use in breeding stocks of falcons. Therefore the immunogenicity and protective capacity of two commercially available WNV vaccines, both approved for use in horses, were evaluated in large falcons. One vaccine contained adjuvanted inactivated WNV lineage 1 immunogens, while the second represented a canarypox recombinant live virus vector vaccine. The efficacy of different vaccination regimes for these two vaccines was assessed serologically and by challenging the falcons with a WNV strain of homologous lineage 1. Our studies show that the recombinant vaccine conveys a slightly better protection than the inactivated vaccine, but moderate (recombinant vaccine) or weak (inactivated vaccine) side effects were observed at the injection sites. Using the recommended 2-dose regimen, both vaccines elicited only sub-optimal antibody responses and gave only partial protection following WNV challenge. Better results were obtained for both vaccines after a third dose, i.e. alleviation of clinical signs, absence of fatalities and reduction of virus shedding and viraemia. Therefore the consequences of WNV infections in falcons can be clearly alleviated by vaccination, especially if the amended triple administration scheme is used, although side effects at the vaccination site must be accepted.

## Introduction

West Nile virus (WNV) is a *Flavivirus* belonging to the family *Flaviviridae* and is encoded by a positive sense, single stranded RNA genome. The virus is distributed worldwide [1]. Whereas until the mid 1990s WNV disease was perceived as sporadic and mild in humans and horses, larger scale epidemics were discovered in subsequent years [2]. In birds WNV was first isolated in Egypt in 1953 [3]. However major outbreaks amongst domestic birds occurred in Israel in 1997 and thereafter. Besides also wild birds were affected [4,5]. In 1999, WNV was introduced to New York City and spread from there to almost the whole American continent. WNV is transmitted between birds by mosquitoes, especially *Culex* species, in an enzootic transmission cycle [6]. Passeriformes and also raptor species are highly susceptible, develop high virus titers and can succumb to the disease [7]. Natural disease producing WNV lineage 1 infections have been described in falcon species including gyrfalcon (*Falco rusticolus*), peregrine falcon (*F. peregrinus*), prairie falcon (*F. mexicanus*), merlin (*F. columbarius*) and American kestrel (*F. sparverius*) [8,9].

Fatal WNV lineage 2 infection has been reported during the epidemic in Austria in one gyrfalcon [10]. The susceptibility of various raptor species has been confirmed in experimental WNV infection studies. Large falcons are susceptible to WNV [11] and developed subclinical to fatal disease as competent amplifying hosts after experimental infection with WNV [12]. Subunit vaccines, inactivated vaccines and DNA vaccines against WNV infections were used with variable success in birds. Vaccine efficacy in these studies was determined by experimental WNV challenge of the birds [13-21] or by determination of neutralizing antibody response [22-24].

Birds of prey, especially falcons, are bred in captivity for species conservation and commercial purposes. As WNV infections in these breeding flocks may cause substantial losses, the availability of an efficacious vaccine with minimal side effects is desirable. However, only very few data on vaccine efficacies in raptor species are available. After vaccination with a DNA vaccine, California condors developed neutralizing antibody responses but no challenge experiment was carried out [22]. Such a vaccination/challenge study in raptors has only been done once using a DNA vaccine in red-tailed hawk [19]. Although the seroconversion rate following a two dose regimen was low (only 3 of 14 hawks developed a detectable yet low antibody titre) and viremia was significantly reduced, virus shedding was not affected. However, none of the WNV infected hawks in the control group showed any clinical signs, which certainly limits the interpretation of this study.

In the study presented herein, two commercially available WNV vaccines for equines were evaluated, namely Duvaxyn® WNV, Fort Dodge (Fort Dodge Animal Health Limited, Hedge End, Southhampton, England; in the EU now Equip® WNV, Zoetis Belgium SA, Louvain-la-Neuve, Belgium; in the US WEST NILE-INNOVATOR®, Zoetis Inc., Florham Park, NJ, USA) and RECOMBITEK®- Equine rWNV vaccine, Merial Limited, Duluth, USA (now approved in the EU as Proteq West Nile, Merial, Lyon, France). Duvaxyn® was used before in avian species [23,25], including raptors (peregrine falcon, American kestrel and red-tailed hawk). Three immunizations with this vaccine resulted in low antibody titers in 60% of the animals, but again challenge experiments were not conducted [26]. In another study red-tailed hawks failed to develop detectable antibodies after immunization with this vaccine [27]. RECOMBITEK® was previously tested in Western scrub-jays (*Aphelocoma californica*) and provided partial protection, but also caused considerable side effects [20]. The present study first estimates the efficacy of these two WNV vaccines in large falcons confirmed by live virus challenge.

## Materials and methods

### Animals

The animals used in this study were 29 captive-bred mature (> 6 months old) gyrfalcons and hybrid falcons (*F. rusticolus* × *F. cherrug* and *F. rusticolus* × *F. peregrinus*). Birds were clinically healthy (clinical examination and endoscopy) and were dewormed prior to the vaccination trial. Susceptibility to WNV infection was determined by serologic assays that were negative for WNV and a closely related (cross-reactive) European flavivirus, Usutu virus.

### Vaccines/vaccination

The first vaccine used in this study was a commercially available inactivated WNV vaccine approved in the EU for use in horses, named Duvaxyn® WNV, Fort Dodge (in the EU now Equip® WNV, Zoetis; in the US WEST NILE-INNOVATOR®, Zoetis). It contains the formalin inactivated whole WNV VM-2, which is a North American isolate belonging to lineage 1 [28]. The second vaccine used in this study was a recombinant live vaccine, also approved for use in horses (RECOMBITEK®- Equine rWNV vaccine, Merial, US, approved in the EU as Proteq West Nile). It consists of recombinant live canarypox virus ALVAC which co-expresses WNV prM and E proteins of a NY’99 isolate (lineage 1). During the vaccination phase with Duvaxyn® birds were held in a hexagonal 125 m^2^ sized and 6 m high aviary in a breeding centre for falcons. Animals vaccinated with the Recombitek® vaccine were kept in an aviary at FLI, Isle of Riems, under safety level 2 conditions (as this genetically engineered vaccine was not approved in Europe at the time). Falcons were fed commercial one-day-old chicks and provided with water ad libitum.

Falcon groups 1 (*n* = 5, Duvaxyn® WNV) and 3 (*n* = 5, RECOMBITEK® West Nile) were vaccinated using a full dose (1 mL) intramuscular (doses split evenly and inoculated into both sides of pectoral muscles). Booster injections were given four weeks post vaccination (wpv) and the animals were challenged eight weeks after the first vaccination (for vaccine groups see Table 1). Groups 2 (*n* = 5, Duvaxyn® WNV) and 4 (*n* = 5, RECOMBITEK^®^ West Nile) received two booster injections 3 and 6 wpv, and were challenged eight weeks after the first vaccination. Group 5 (*n* = 8) served as non-vaccinated control group. These birds were subdivided and 2 falcons per infection group were handled and housed together with the vaccinated individuals. One bird served as non-vaccinated, non-challenged control referred to as group 6 (*n* = 1) and was housed together with animals of group 3. During the course of vaccination the health status of the falcons was assessed daily, and every week a clinical examination was conducted as described later with additional palpation of the vent, auscultation of heart and lungs, inspection of nares, pharynx, mucous membranes, feet, skin and muscles at the vaccination sites. Blood samples as well as swabs from oropharynx and cloaca were taken weekly. These samples were used for WNV antibody determination by ELISA along with VNT and for WNV as well as recombinant vaccine genome detection, respectively. During these procedures birds were anesthetized with isoflurane inhalation anaesthesia.

**Table 1:** Viral load of organs in tissue culture infectious dose 50 (TCID50) and genome copies

**Group**	**Bird**	**dpi**	**Brain**	**Spleen**	**Kidney**	**Lung**	**Liver**	**Heart**
			**TCID50/mg/copies/mg**	**TCID50/mg/copies/mg**	**TCID50/mg/copies/mg**	**TCID50/mg/copies/mg**	**TCID50/mg/copies/mg**	**TCID50/mg/copies/mg**
**Group 1**	F14	21	0/0	0/0	0/0	0/0	0/0	0/0
**Inactivated**	F15	19	0/4.6	0/0.4	0/0.9	0/0	0/0.1	0/0
**Boost: 4 wpv**	F16	20	0/24.8	0/3.8	0/143	0/0	0/0	0/1.8
**Challenge: 8 wpv**	F17	9	18.7/2530	0/3441	0.2/4479	0/6583	0/290	1164/38565819
	F18	21	0/4.7	0/0.9	0/223	0/0	0/0.1	0/2.6
**Group 2**	F37	19	0/0	0/0	0/0	0/0	0/0	0/0
**Inactivated**	F38	20	0/0	0/0	0/0	0/0	0/0.2	0/0
**Boost: 3 + 6 wpv**	F40	19	0/0.6	0/0	0/0	0/0	0/0	0/0
**Challenge: 8 wpv**	F41	20	0/0	0/1.4	0/0	0/0	0/0	0/0
**Group 3**	F19	20	0/0	0/0	0/0.6	0/0	0/0	0/0
**Recombinant**	F20	19	0/0	0/0	0/0	0/0	0/0	0/0
**Boost: 4 wpv**	F21	21	50.8/4337	0/0	0/141	0/0	0/0	0/0
**Challenge: 8 wpv**	F22	21	0/0	0/0	0/0	0/0	0/0	0/0
	F23	20	0/0	0/0	0/0	0/0	0/0	0/0
**Group 4**	F44	20	0/6	0/0.1	0/0	0/0	0/0	0/0
**Recombinant**	F45	19	0/0	0/0	0/0	0/0	0/0	0/0
**Boost: 3 + 6 wpv**	F47	21	0/0	0/0	0/0	0/0	0/0	0/0
**Challenge: 8 wpv**	F48	19	0/0	0/0	0/0	0/0	0/0	0/0
**Group 5**	F13	19	0.6/112	0/17.9	0/3.9	0/0.1	0/0.3	0/4.6
**Control**	F24	20	0/32.1	0/0	0/822	0/0	0/0	0/4.7
**No vaccination**	F27	14	8.7/132202	0/42	0/403	0/36.9	0/16.7	37.9/7794
	F36	20	0/23.6	0/0	0/2	0/0	0/0	0/0
	F42	5	46.9/515	175/318323	76.2/14400	601/5456	94.4/32119	19/6563
	F43	20	0.4/38.2	0/11.4	0/1.5	0/0	0/0.1	0/0.1
	F51	8	738/2668	10.7/8631	2896/82091	7558/63844	3023/402895	4380/295311
	F55	3	0/12.3	3.3/2376	3.9/796	13.2/465	0.9/249	5.4/380
**Group 6**	F63	21	0/0	0/0	0/0	0/0	0/0	0/0
**Env. Control**								

*Abbreviations:* copies = genome copies; dpi = days post infection, Env. = environmental, wpv = weeks post vaccination.

Values are given per mg tissue. High TCID50 were found in birds that died after infection, whereas virus was scarce or absent in birds that survived the whole three-week challenge period. Sensitivity of qRT-PCR was generally higher than virus titration in cell culture. Statistical analysis of viral load was performed for brain, spleen, kidney and heart. There is a statistically significant difference to group 5 in brain, kidney and heart of group 2; spleen, kidney and heart of group 3 and in brain, kidney and heart of group 4.

Animal vaccination and challenge experiments described in this manuscript were approved by the competent authority of the Federal State of Mecklenburg-Western Pomerania, Germany (reference number 7221.3–1.1.-056/10) on the basis of national and European legislation, namely EU council directive 86/609/EEC for the protection of animals used for experiments.

### Nested PCR for detecting recombinant vaccine shedding

Oropharyngeal and cloacal swab samples were placed in sterile polypropylene tubes filled with 2 mL of minimal essential medium (MEM) supplemented with antimicrobial drugs and stored at −70 °C for further examinations. Viral DNA was extracted from swab sample supernatants using the QIAamp DNA Kit (QIAGEN GmbH, Hilden, Germany) according to the manufacturer’s instructions. The PCR used the following primers (fw CTCGTTAATTAATTAGAGCTTC and rev CAATGCATAGGTTCTTTCCAGC) binding in the ALVAC vector and in the WNV prM gene, respectively [29]. PCR was performed using the Fermentas Dream Taq^TM^ DNA Polymerase (Thermo Fisher Scientific Inc., Waltham, MA, USA) and the following temperature profile: 95 °C for 3 min and 40 × 95 °C for 30 s, 47.8 °C for 30 s, 72 °C for 1 min and then 72 °C for 15 min. The nested PCR used the internal primers (nfw CAAAGGTTCTTGAGGGTTGTG and nrev GTTGGAATCGTGATGACATCTG) and the following temperature profile: 95 °C for 3 min and the 40 × 95 °C for 30 s, 52.9 °C for 30 s, 72 °C for 1 min and 72 °C for 15 min. The commercial vaccine virus RECOMBITEK® served as positive control. Spike experiments showed no inhibitory effects of falcon oropharyngeal and cloacal swabs, and serial dilution series revealed a sensitivity of detection of ≈ 170 copies/PCR reaction (based on the canarypox genome size).

### WNV challenge of the falcons

WNV lineage 1 NY99 was used for challenge experiments (GenBank accession no. AF196835, virus identity verified by nucleotide sequence encoding for E protein). Virus was propagated on Vero E6 cells (FLI, Greifswald, Germany) and virus titers were determined. Challenge virus doses diluted in MEM contained 10^6^ Tissue Culture Infection Dose 50 (TCID_50_) in 1 mL. All WNV infection studies were carried out under biosafety level 3 conditions. Two vaccinated groups (group 1 + 3 or group 2 + 4, respectively), two non-vaccinated birds of group 5 and one non-vaccinated, non challenge control bird (group 6) were kept together in one room.

Animals were caged separately and fed commercial one-day-old chicks ad libitum (maximum eight chicks per day). Infections were carried out subcutaneously in the left inguinal region under isoflurane inhalation anaesthesia [30].

Following infection all falcons were examined daily for the following criteria, which were summed up using a clinical score system: general condition, posture, plumage, behaviour, excrements, neurological status, hydration status, respiration and food uptake. A clinical score was determined by adding 0 to 3 points for deviation in each criterion from normal state (−, +, ++, +++) to a total sum. Four clinical score classes were defined as follows. Clinical score of 0 = healthy (0 to 0.5 deviation points), 1 = mildly affected (1 to 4.5 deviation points), 2 = moderately affected (5 to 10.5 deviation points), 3 = severely affected (11 to 24 deviation points) and 4 = death. In parallel to sampling, temperature, body weight, body condition score and hydration status were measured under isoflurane inhalation anaesthesia. Sampling included blood samples, oropharyngeal and cloacal swabs and was performed on 0, 3, 6, 8 (only swabs), 10, 12 (only swabs), 14 and 19/20/21 days post infection (dpi) (or earlier in case of euthanasia). Blood was diluted in bovine albumin-1 (BA-1) diluent [31] immediately after sampling and stored at −70 °C until further analysis. Swabs were transferred to 2 mL MEM supplemented with antibiotics (1 g/L enrofloxacin, 0.5 g/L spectinomycin, 0.25 g/L lincomycin and 0.05 g/L gentamycin) and shaken for 30 min at room temperature. Dead animals were examined pathologically and organ samples (brain, spleen, kidney, lung, liver, and heart) were fixed in formalin or stored in supplemented MEM at −70 °C.

### Virus isolation

Organ samples in supplemented MEM were homogenized in a tissue lyser bead mill (Qiagen). Serial dilutions of organ-homogenate supernatants and of BA-1 blood dilutions were used to inoculate Vero cells. After seven days of incubation, cells were formalin-fixed, stained with crystal violet and virus titers calculated using the Spearman and Kaerber method [32].

### Serological assays

After centrifugation, sera were assayed by ID Screen© WN competition ELISA (IDVet, Grabels, France) following the manufacturer’s instructions.

Neutralizing WNV antibody titers were determined by VNT on Vero cells as published previously [33] using 1:10, 1:20, 1:40 etc. dilutions (in MEM) and homologous challenge virus (100 TCID_50_ of WNV lineage 1 NY99, GenBank accession no. AF196835). Assays were read after seven days after formalin-fixation and crystal violet staining of the cell monolayers. Cytopathogenic effects were measured and neutralizing titres were calculated using the Behrens-Kaerber method [34]. Neutralization test for Usutu virus (USUV) was performed under the same conditions using USUV strain Vienna 2001 (GenBank accession no. AY453411.1).

### Quantitative real-time RT-PCR (qRT-PCR)

Viral RNA was isolated from swab samples and organ homogenate supernatants using the QIAamp® Viral RNA Kit (Qiagen). Viral RNA of blood in BA-1 medium was extracted using the RNeasy® Mini Kit (Qiagen) following the manufacturer’s instructions including a TRIzol® (Life Technologies, Carlsbad, CA, USA) chloroform step. An internal control RNA (IC RNA) containing 2 × 10^5^ copies/μL was extracted together with all samples, which were then stored at −70 °C.

Two previously published qRT-PCRs which target either the 5′NTR-region or the NS2A-region were used [35]. Viral loads were determined based on cycle threshold (CT) values and in case of organ samples quantified as copies/μL RNA using an external calibrator control [35].

### Histopathology/Immunohistochemistry (IHC)

Tissue samples were fixed in neutral buffered formalin (4%), embedded in paraffin, 3 μm sections were cut and stained with haematoxylin/eosin (HE). For IHC 3 μm sections were cut, deparaffinised and rehydrated. As primary antibody mab 15R4 (kindly provided by Petra Emmerich, Bernhard-Nocht Institute, Hamburg, Germany) was used at a dilution of 1:20 in goat serum (FLI). To verify inconclusive results mab 3B2 (kindly provided by Davide Lelli, Istituto Zooprofilattico Sperimentale della Lombardia e dell’Emilia Romagna, Brescia, Italy) was used at a dilution of 1:80 in goat serum. Endogenous peroxidase was blocked by 3% H_2_O_2_/methanol incubation and antigen was retrieved by Proteinase K digestion (15 min at 37 °C with 4 μg/mL). On sections incubated with mab 3B2 high-temperature citrate buffer (pH 6.0, microwave for 10 min) was additionally applied. As secondary antibody Mouse Envision HRP (Dako Diagnostics, Dako Deutschland GmbH, Hamburg, Germany) and as substrate diaminobenzidine were used.

Brains, hearts, spleens, kidneys and the virus injection sites were evaluated. Depending on the proportion of positive tissue in the slide, the organ was scored from 0 to 3 (0 = no positive tissue = negative, 1 = < 1% positive tissue = mildly affected, 2 = ≥ 1% and < 5% positive tissue = moderately affected, 3 = ≥ 5% positive tissue = severely affected).

### Statistical analysis

All statistical tests were conducted using R software [36]. Always one group was tested independently against the control group. *P*-values below α-levels of 0.05 were considered statistically significant. After addition of clinical scores of all days for each falcon they were compared using one-sided Wilcoxon rank sum test with continuity correction. For 19, 20 and 21 dpi the arithmetic means of these 3 days were used. The same applies to mean duration time of illness. Duration of illness was defined as the sum of all days one falcon had a clinical score of 1 to 3. A clinical score of 4 was not included in duration of disease. Variance analyses for oral and cloacal shedding levels and viraemia levels were conducted by ANOVA over all sampled days. The duration of shedding and viraemia was evaluated by the one-sided Wilcoxon rank sum test with continuity correction by using a cut off Ct value of 35.0 as negative in qRT-PCR. For analysis of viral load in organs (brain, spleen, kidney, heart) the values of all surviving falcons were evaluated by one-sided Wilcoxon rank sum test with continuity correction.

## Results

### Vaccination

A total of 20 falcons were vaccinated with either a formalin inactivated WNV vaccine (Duvaxyn®) or a recombinant canarypox live WNV vaccine (RECOMBITEK®) twice (groups 1 and 3) or three times (groups 2 and 4), respectively. Prior to vaccination all falcons were seronegative for WNV (ELISA, VNT) and USUV (VNT) specific antibodies.

After vaccination no clinical side effects were observed in groups 1, 2 (Duvaxyn®) and 4 (RECOMBITEK® three times) at any time of the study. Falcons (5/5) in group 3 (RECOMBITEK® twice), however, showed an unexplained interim reduction of body weight (of up to 20% and more). Moreover, in this group local inflammations at the injection sites (pustules, induration and slight swelling) were observed in 3/5 birds (RECOMBITEK® two times), but oral and cloacal shedding of recombinant canarypox viruses was not observed, since swabs were negative for the ALVAC/WNV-prM genome by nested PCR.

**Figure 1:**
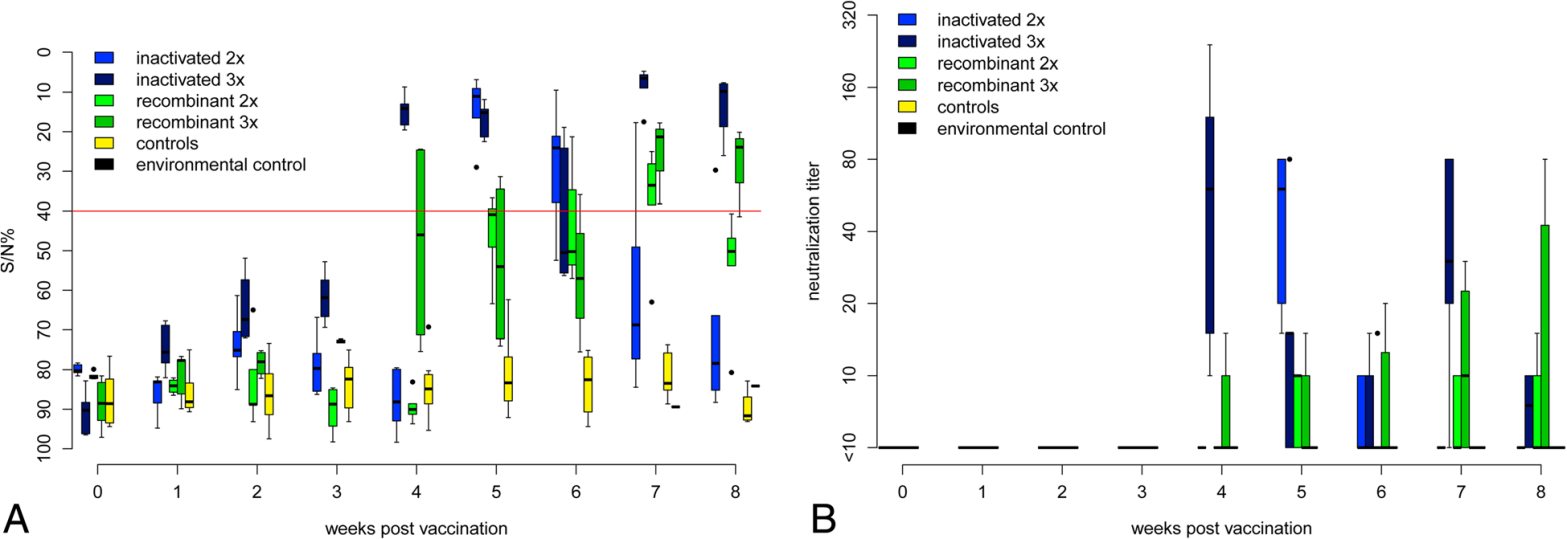
**Antibodies in falcons detected by ELISA and micro-virus neutralization test (VNT) during vaccination period.** Figure 1**A** shows competition ELISA (ID Screen© WN competition ELISA, IDVet, Grabels, France) responses. Optical density at 450 nm is converted to signal/noise% (S/N%) ratio (S/N% = OD_sample_/OD_negative control_* 100), with values ≤ 40% considered positive, > 40% and ≤ 50% equivocal and > 50% negative. The threshold for positive results is indicated as a solid red line. Figure 1**B** displays neutralization antibody responses for all groups. Data are presented in a box-and-whisker plot, where the ends of the whiskers represent the minimum and maximum values, respectively. Outliers are represented as points instead of whisker-ends. The box includes 50% of the values of each group and the line in the middle of each group represents the median value of each group. The double immunization with inactivated vaccine (light blue) led to temporary seroconversion whereas the triple vaccination (dark blue) was more efficient. The recombinant vaccine generally induced only a slight seroconversion. With two shots of the recombinant vaccine (light green) low level and temporary seroconversion occurred, while three shots (dark green) provided a better result measureable by ELISA but not by VNT.

Two injections of Duvaxyn® (group 1) stimulated a low antibody response by week 5 post vaccination (ELISA positive results and VNT levels of 15 up to 80), but these antibodies disappeared in 4/5 falcons until week 8 (Figures 1A and B). However, three consecutive injections of this inactivated vaccine (group 2) induced a more robust antibody response and an abiding seroconversion in ELISA in all (5/5) falcons. Neutralizing antibody titers in these birds reached 240 and lasted in 2/5 birds until the challenge date in week 8.

Antibody responses induced by the RECOMBITEK® vaccine (groups 3 and 4) were generally lower than those induced by the inactivated vaccine (group 1 and 2).

Two canarypox vector vaccine applications induced antibodies in 5/5 birds which disappeared in 3/5 falcons. Maximum neutralizing titers were 15. Three vaccine applications raised ELISA detectable antibodies in 3/4 birds at the end of the vaccination phase (8 wpv) and 1/4 falcons developed a neutralizing antibody titre of 80.

Two falcons (F39, 8 wpv, inactivated group 2 and F46, 7 days post vaccination (dpv), recombinant group 4) died suddenly for reasons most likely unrelated to the vaccination (severe visceral gout and severe intestinal endoparasitosis, respectively) and were therefore excluded from the statistical analysis.

### Challenge studies

#### Non-vaccinated control birds (NC - group 5)

Following WNV challenge all (8/8) non-vaccinated falcons developed moderate to severe clinical signs including impaired general condition, crouched body postures, ruffled feathers, apathy, inappetence, dehydration and a greenish discoloured uric acid component of the excrements. All birds lost body weight – in some animals up to 20% and more. Neurological symptoms such as tilted head, ataxia, seizures and recumbency were observed in two birds. Overall four of the eight non-vaccinated WNV challenged birds died or had to be euthanized for animal welfare reasons on 3, 5, 8, and 14 dpi eventually. Mean duration of clinical symptoms was 8.6 days (Additional file 1) with at least moderate disease (clinical score of 2), in one bird severe disease (clinical score of 3).

**Figure 2:**
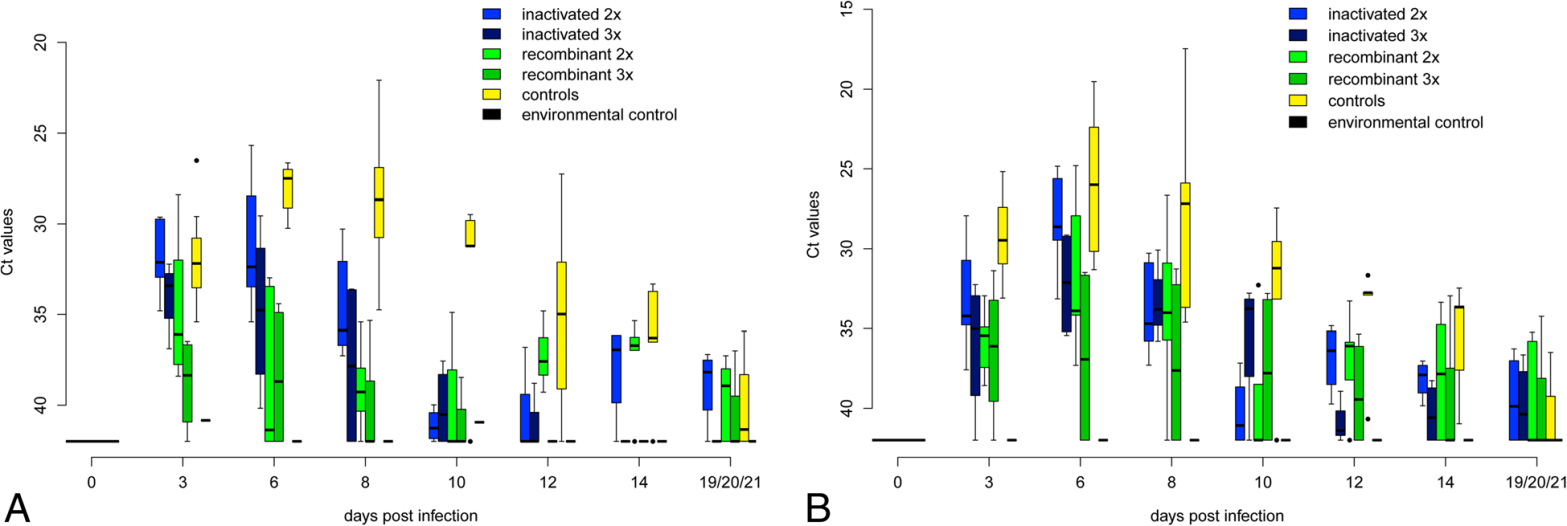
**Oral and cloacal shedding of falcons during challenge.** Cycle threshold values (Ct values) of oral **(A)** and cloacal **(B)** swabs for all groups are displayed. Data are presented in a box-and-whisker plot, where the ends of the whiskers represent the minimum and maximum values, respectively. Outliers are represented as points instead of whisker-ends. The box includes 50% of the values of each group and the line in the middle of each group represents the median value of each group. The amount and duration of oral shedding was significantly reduced in the group vaccinated three times with Duvaxyn® (dark blue, only amount reduced) and in the groups vaccinated with Recombitek® (light and dark green), but not in the group vaccinated twice with Duvaxyn® (light blue). In addition, the amount of cloacal shedding was significantly reduced in all groups. Duration of cloacal shedding was not shortened in any of the groups.

**Figure 3:**
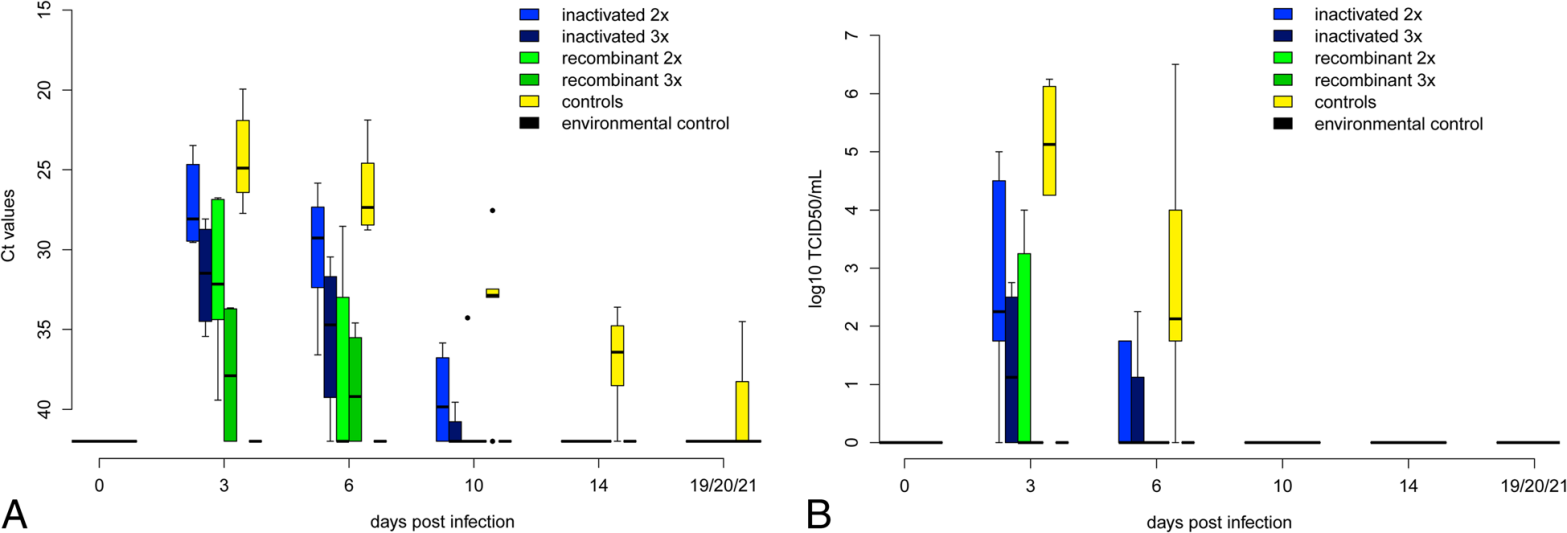
**Viraemia detected by qRT-PCR and virus titration.** Cycle threshold values (Ct values) of whole blood for all groups during the challenge period are displayed in Figure 3**A**. Results of blood titration for all groups during the challenge period are given in Figure 3**B** in log_10_ tissue culture infection dose 50 per mL (TCID_50_/mL) whole blood. Data are presented in a box-and-whisker plot, where the ends of the whiskers represent the minimum and maximum values, respectively. Outliers are represented as points instead of whisker-ends. The box includes 50% of the values of each group and the line in the middle of each group represents the median value of each group. The level of viraemia measured in Ct values was significantly lower in all vaccinated groups than in the control group (yellow). In contrast, duration of viraemia was only shortened significantly in the group vaccinated three times with Recombitek® (dark green).

**Figure 4:**
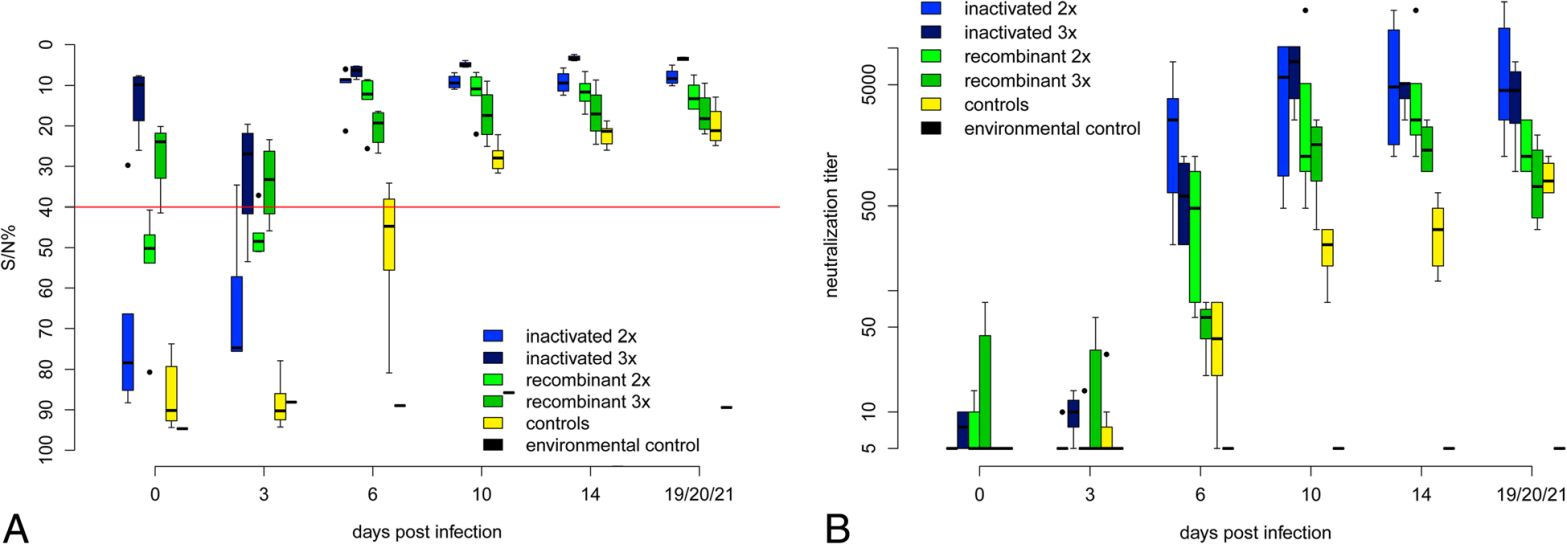
**Antibodies detected by ELISA and micro-virus neutralization test (VNT) during challenge in falcons.** In Figure 4**A** antibodies of all groups detected by the ID Screen© WN competition ELISA (IDVet, Grabels, France) during the challenge period are displayed. Optical density at 450 nm is converted to signal/noise% (S/N%) ratio (S/N% = OD_sample_/OD_negative control_ * 100), with values ≤ 40% considered positive, > 40% and ≤ 50% equivocal and > 50% negative. The threshold for positive results is indicated as a solid red line. In Figure 4**B** antibody titers of all groups determined by VNT against homologous challenge virus (neutralization titer) during the challenge period are displayed. Data are presented in a box-and-whisker plot, where the ends of the whiskers represent the minimum and maximum values, respectively. Outliers are represented as points instead of whisker-ends. The box includes 50% of the values of each group and the line in the middle of each group represents the median value of each group. Seroconversion occurred at 3 to 6 dpi.

In these non-vaccinated control animals virus shedding occurred from 3 to 14 dpi with an initial peak from 3 to 8 dpi (Figures 2A and B). Highest virus loads in oral and cloacal swabs reached Ct values of 22.1 and 17.5, respectively. Viral RNA was detected in the blood from 3 to 21 dpi with a peak during the first days (lowest Ct value 19.9) (Figures 3A and B). Virus loads in the organs of the deceased or euthanized falcons were high (Table 1). Copy numbers and Ct values of the organs of non-vaccinated birds reached 402 895 cop/mg and 16.7, respectively. All birds (except two which died early in the course of the experiment after 5 and 8 dpi, respectively) seroconverted after 3 to 6 dpi (Figures 4A and B).

All falcons were necropsied either after death, euthanasia or at the end of the observation period (21 dpi). One of the eight animals died 3 days post challenge with severe acute hemorrhagic enteritis and clear signs of septicaemia and was therefore excluded from histopathological analysis. However, molecular and immunohistochemical studies showed that it was successfully infected (see below). The seven remaining animals had pale and up to moderately enlarged spleens and pale myocardial foci. Myocardial petechiae were observed in one falcon which had died after 5 dpi. Lesions seen histopathologically associated to a WNV infection are mild to moderate non-suppurative encephalitis (3/7) or meningo-encephalitis (4/7) and mild to moderate acute or subacute lymphohistiocytic necrotizing myocarditis (5/7). Non-suppurative necrotizing arteritis was seen in the spleen of one bird only. All other findings, affecting in particular spleen and liver are due to more unspecific reactions. Detailed histopathological results are shown in the supplemental data (Additional file 2).

**Figure 5:**
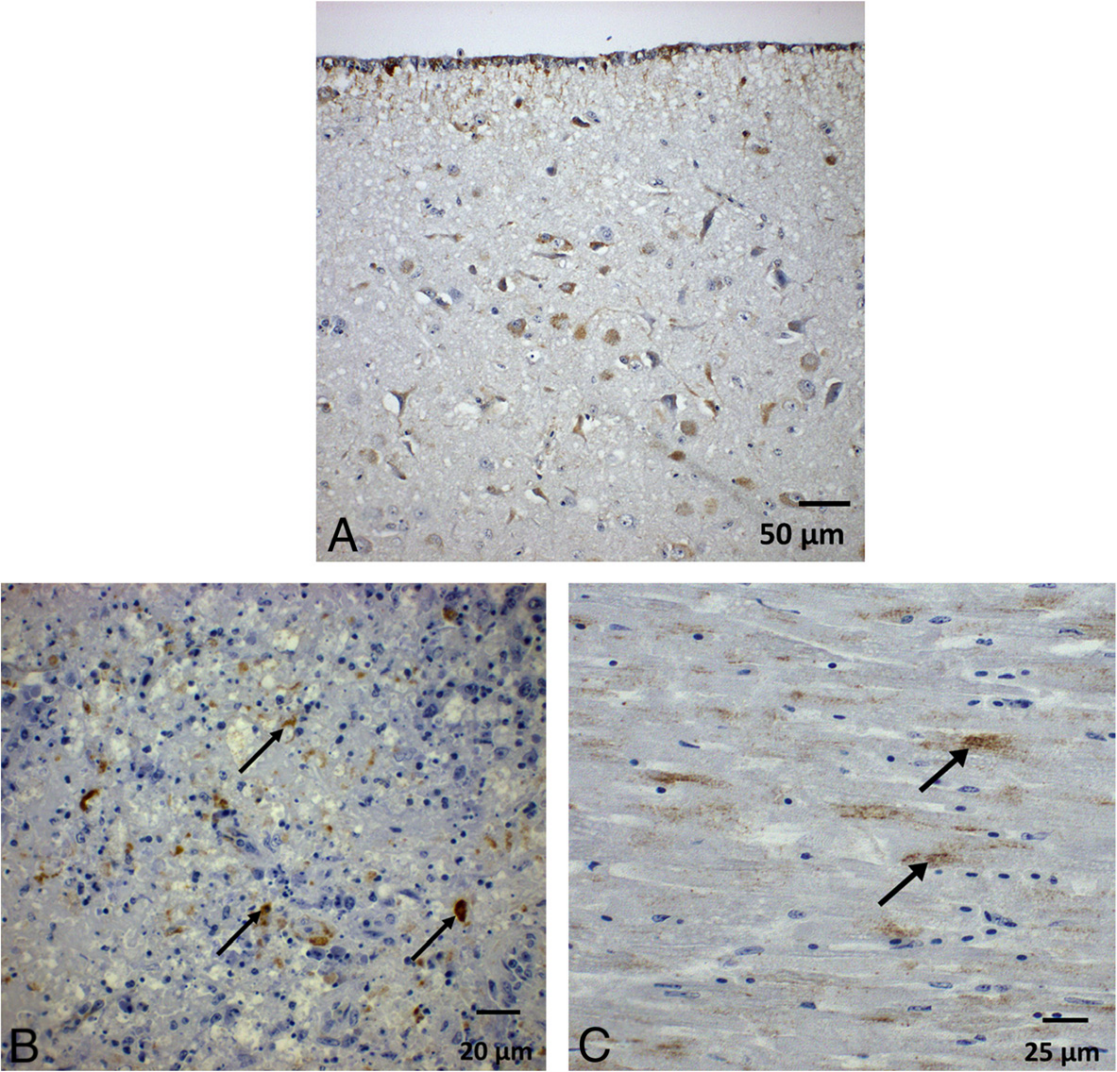
**WNV antigen dectection by immunohistochemistry in different tissues of falcons after virus challenge. A**: Brain/Telencephalon (region lateral of the lateral ventricle) of a challenged falcon (F6) included in a previously published study [12] at day 10 post infection. **B**: Spleen of a bird at day 8 post infection without vaccination (F51). Antigen was detected in necrotic material of follicle arteries. **C**: Myocard of a falcon vaccinated twice with the inactivated vaccine (F17, necropsied at day 9 post infection). Antigen staining in cardiocytes (arrows). All results were obtained using the monoclonal antibody 15R4 (kindly provided by Petra Emmerich, Bernhard-Nocht Institute, Hamburg, Germany).

Immunohistochemical examination of the brains, hearts, spleens and injection sites revealed differences between the individual animals. WNV antigen was found in distinct quantities in the fatally diseased animals (4/8), whereas only very scant (2/8) or no (2/8) WNV antigen staining was detected in challenge survivors. In order to demonstrate a large WNV antigen deposition, IHC staining results of another fatally affected falcon (F6) from an earlier experiment [12] are reported here for comparison. Figure 5A-C shows the immunohistochemical reaction pattern in different tissues, a detailed description can be found in the supplemental data (Additional file 2).

#### Environmental control bird (group 6)

The non-vaccinated, non-infected control bird (F63) did not develop any clinical signs during the challenge period, and no WNV RNA and no WNV-reactive antibodies were detected in swab, blood and organ samples.

#### Inactivated vaccine - two dose regimen (Duxaxyn® - group 1)

Falcons immunized twice with the inactivated vaccine suffered from clinical infection for 7.6 days on average and one falcon died after 9 dpi. Four out of five birds showed signs of moderate illness (clinical score of 2; Additional file 1) over several days and one bird developed only mild disease (clinical score of 1). However, clinical scores were significantly lower (*p* value 0.01677, Additional file 3) than those of the non-vaccinated falcons. Vaccinated falcons shed WNV orally (3 to 8 dpi/lowest Ct value of 25.7) and cloacally (3 to 12 dpi/lowest Ct value of 24.8) for the same time period as the non-vaccinated animals (*p* values 0.1013 and 0.1663). However, the extent of cloacal shedding (*p* value of 0.0001274) but not of oral shedding (*p* value 0.06382) was significantly reduced in comparison to group 5.

All birds in this group developed viraemia (from 3 to 6 dpi). While the duration of viraemia was not significantly reduced (compared to group 5; *p* value 0.1566), viraemia levels were significantly lower (lowest Ct value of 23.5, *p* value 4.468e-05). Copy numbers in brain, spleen, kidney and heart at 21 dpi were similar to those in non-vaccinated falcons (*p* values > 0.1286). The one falcon which died (F17) had very high viral loads, especially in the heart, with a Ct value of 11.7 (details Table 1). The remaining birds showed evidence of viral infection in organs; especially in the kidney (Ct values to 27.9). Nonetheless 1/5 birds (F14) was completely negative in all organs.

Organs of all surviving birds were virus negative in cell culture (kidney, brain and heart of F17 were positive).

Seroconversion was observed in 2 birds by 3 dpi and in all birds by 6 dpi as determined by ELISA and VNT. Maximum VNT titers ranged from 640 to 24 000 eventually.

One bird (1/5, F15) displayed only mild phlebitis at the vacciniation site. All other birds (4/5) showed acute, nonsuppurative encephalitis of varying degress, in one case (F16) with involvement of the meninges. Additionally, acute to subacute necrotizing myocardial lesions of varying degrees (4/5) and mycordial petechiae (1/5) were present. The pale and enlarged spleen of all falcons showed nonspecific signs such as severe lymphoid depletion with marked lymphocytolysis in the few remaining follicles (3/5) and infiltration of bloated foamy macrophages (5/5). In immunohistochemistry 2/5 birds had antigen in different organs. In the fatally diseased animal (F17) antigen was detectable in all examined organs, especially in the heart (positive reactions in > 75% of the myocardium, Figure 5C).

#### Inactivated vaccine - three dose regimen (Duvaxyn® - group 2)

Following three immunizations falcons developed only mild disease (maximum clinical score of 1, i.e. significantly reduced clinical scores compared to non-vaccinated controls; *p* value 0.00418) for 6.25 days on average. However, oral and cloacal shedding and viraemia still occurred in the animals of this group, albeit only at very low levels (oral shedding on days 3 to 8, lowest Ct value 29.6/cloacal shedding on days 3 to 10, lowest Ct value 29.1/viraemia days 3 up to 6, lowest Ct value 28.1). The amount of oral and cloacal shedding and viraemia was significantly reduced compared to non-vaccinated birds (*p* values of 1.269e-08, 2.006e-06 and 3.43e-08). Duration of oral and cloacal shedding and viraemia was not shortened (*p* values > 0.06). No virus was detectable in any organ at the end of the study and viral RNA was detectable in low amounts in 3/4 animals (Ct values > 35.0) in single organs (statistically significant difference to control group for brain, kidney, heart compared to negative control group 5; *p* values 0.0128, 0.0128, 0.02953; for spleen 0.1125). All 4/4 birds elicited neutralizing antibody titers with peak titers ranging from 5120 to 10240.

One bird (F41) developed lymphocytolysis of locally aggregated follicles at the vaccination site. However histopathological findings in some of the birds were typical for WNV infections. F38 displayed acute nonsuppurative encephalitis and necrotizing myocarditis. F40 had a weak encephalitis combined with local lymphohisticytic inflammation at the vaccination site and F37 showed necrotizing arteriitis in connective tissues (skin, parenchymas) and in liver which was associated with acute necrosis of hepatocytes and mixed cell infiltration. Additionally, extensive PALS necrosis with fibrin deposition and involvement of giant cells was seen in the spleen. All bird tissues which were examined by IHC at 21 dpi gave negative results.

#### Recombinant canarypox vector vaccine two dose regimen (Recombitek® - group 3)

Falcons vaccinated twice with the recombinant canarypox vector vaccine developed moderate disease (maximum clinical score of 2), including one bird showing only mild disease (clinical score of 1; mean duration 7.8 days; in comparison to non-vaccinated group *p* value 0.005155). Compared to the non-vaccinated controls virus shedding by the oral and cloacal route was significantly reduced (max. Ct values of 28.4 and 24.8 respectively; *p* values 9.710e-07, 2.385e-05) and oral shedding ceased earlier (12 dpi, *p* value 0.03158; cloacal shedding 14 dpi, *p* value 0.1442). Indeed 3/5 animals showed only borderline qRT-PCR results for oral shedding (Ct values > 35.0). Viraemia was detected in all birds at significantly lower levels (lowest Ct level 26.8, *p* value 1.025e-07), but lasted as long as in the non-vaccinated animals (*p* value 0.07688).

Single organs of 2/5 birds in this group were qRT-PCR positive, e.g. one brain (F21) with a Ct value of 26.9 being the only organ with a positive titer in cell culture. Viral loads in spleen, kidney and heart were lower than in the control group (*p* values 0.02228, 0.01063, 0.008443; for brain 0.0561).

At 6 dpi all birds in group 3 seroconverted (maximum titers from 9360 up to 20480). All falcons displayed extensive granulomatous myositis at the vaccination site. Only mild histopathological findings associated with WNV infection were seen in 4/5 birds and included nonsuppurative (meningo)encephalitis (3/5) and subacute necrotizing myocarditis (3/5). Nonspecific signs in spleen and liver, as described above for the control falcons, were seen in all animals.

Weak WNV antigen staining was found by IHC in 2/5 birds (in spleen and in heart and diencephalon respectively).

#### Recombinant canarypox vector vaccine three dose regimen (Recombitek® - group 4)

Three vaccinations with the recombinant vaccine led to very mild WNV associated disease in 4/4 animals (maximum clinical score of 1; compared to the non-vaccinated birds statistically significant with a *p* value 0.00418, mean duration 3.75 days).

Oral shedding was observed in only 1/4 birds (6 dpi, Ct value 34.4) and cloacal shedding in 2/4 birds (3 to 21 dpi with peak at 3 to 6 dpi; lowest Ct value 31.3), however 1/4 birds showed borderline qRT-PCR results (Ct values > 35). The amount of oral and cloacal shedding and duration of oral shedding were significantly reduced compared to non-vaccinated group 5 (*p* values 1.040e-11, 1.187e-06, 0.006011; for duration of cloacal shedding 0.1139).

In 2/4 birds viraemia was detected on 3 to 6 dpi (lowest Ct values of 33.7; level and duration significantly reduced compared to group 5 with *p* values of 1.723e-09 and 0.02339).

By qRT-PCR a low level of WNV RNA (Ct > 33.0) was found in one falcon (*p*-values of brain, kidney and heart 0.01377, 0.0128, 0.02953; for spleen 0.1125; comparison data of non-vaccinated birds), but virus could not be re-isolated by cell culture from any of the organs.

All birds seroconverted at 6 dpi reaching maximum neutralization titers from 960 to 2560.

At the vaccination sites extensive granulomatous (4/5 birds) and pyogranulomatous (1/5 birds) myositis was detectable in all animals. 2/5 birds showed moderate (meningo-)encephalitis as well as myocardial alterations such as acute hemorrhage and necrotizing myocarditis. In addition F44 showed mild lymphohistiocytic hepatitis, pancreatitis and neuritis. The spleen was inconspicuous in gross examination (3/4 birds), but lymphoid depletion and lymphocytolysis was seen in two animals. By IHC one bird (F44) was slightly positive in heart.

## Discussion

WNV infections in large falcons can be fatal [12]. So far, there is no WNV vaccine approved for use in any bird species, and only few data on the antibody responses of avian species following the application of commercially available vaccines are available. The aim of the present study was therefore to evaluate the efficacy and safety of two WNV vaccines in large falcons.

The inactivated virus vaccine used in the present study was used in red-tailed hawk before, where it failed to stimulate a detectable antibody response using a two shot regimen and reduced vaccine doses (20% of the full dose) [27]. In another study a three shot full dose regimen led to a detectable seroconversion (NT titers of 10) in 58.3% of birds of prey (including falcons) and corvids, but no challenge studies were conducted [26]. In the present study a higher seroconversion rate and higher maximum antibody titers were achieved in falcons using a similar vaccination scheme. The fact that sampling was conducted at different time points after vaccination limits comparability of both studies. However, the present study is the first one that determines the true efficacy of the vaccine by subsequent WNV challenge studies in a raptor species.

The immunogenicity of the canarypox vector vaccine has been studied in Western scrub-jay recently, but failed to stimulate antibody responses after single vaccination, and pathological alterations typical for WNV infections and reduced viraemia were observed after WNV NY99 challenge [20]. The absence of antibody responses at two and four wpv in the scrub-jays corresponds to findings in the present study in falcons. In these scrub-jays, vaccine application induced massive necrotic lesions at the application sites (i.e. pectoral muscles) [20]. These findings correspond to results in the present study. In falcons the inactivated virus vaccine was tolerated well (mild non-specific inflammatory response at the injection site in single animals), but the canarypox-based vaccine caused a massive local inflammation in most animals.

Environmental safety is an important issue for this genetically engineered vaccine. As the canarypox vector virus was shed neither orally nor via feces by any of the vaccinated falcons, this concern seemed to be unjustified. However, the host species restriction might not be as tight as expected, since local amplification may have occurred at the injection site. Previous studies have shown that this vector (ALVAC) does not replicate in mammals [37], but is able to replicate in chicken cells in vitro [37].

Two injections of the inactivated vaccine provided only insufficient protection. Animals were in poor clinical condition post challenge and one bird died. Although the amount of cloacal shedding was reduced significantly, the amount of oral shedding and the duration of shedding were not reduced. Viraemia was present in falcons of this group, even though levels were generally lower than those observed in non-vaccinated control falcons. The threshold for infectiveness to *Culex pipiens* mosquitoes is considered to be 10^5^ pfu/mL serum [7] which was still reached using the two shot regimen. Therefore birds could have been infectious for mosquitos.

In comparison, triple immunization with the inactivated vaccine led to much lower clinical scores and virus shedding and also to viraemia levels well below mosquito infectiousness.

In terms of protection against WNV challenge, the live recombinant canarypox based vector vaccine may be regarded as superior to the inactivated vaccine, which is supported by the fact that besides antibody production live vaccines also stimulate cellular immune responses [37]. In the present study two vaccinations with the recombinant vaccine did not prevent clinical disease. Viral shedding and viraemia still occurred, although at a lower level than in non-vaccinated birds, effectively blocking transmission to mosquitoes.

Triple vaccination with the canarypox-based vaccine also failed to completely protect the falcons. However, clinical symptoms were reduced sufficiently and shedding of WN-viral RNA was decreased and in some birds completely eliminated. Additionally, this vaccination schedule provided sterile immunity in two birds and low levels of viral RNA detection among blood and organs.

Independently of the vaccination scheme, specific pathological findings for WNV were found in nearly all animals, such as nonsuppurative (meningo)encephalitis and necrotizing myocarditis of varying degrees [12]. Given the differences in the course of the disease in the single animals, there are also individual/multifactorial factors in addition to the vaccination which had an impact on how the animals overcame WNV challenge. As decribed by others [38-40] lymphohistiocytic inflammations were seen in different parenchymas (liver, kidney, pancreas, peripheral nerves), however, in single animals only. Therefore these inflammations might not be associated with WNV infection directly. Alterations of the spleen were most probably due to nonspecific reactions associated with viral infections (i.e. severe lymphoid depletion and lymphocytolysis of remaining follicles, PALS necrosis) or due to the outcome of the disease, such as the detection of foamy bloated macrophages which are indicative for an altered fat metabolism [41].

Only low amounts of viral antigen were detected by IHC in most animals, most probably due to the fact that most falcons survived the challenge and were necropsied only at the end of the study period. In contrast, distinct IHC reaction patterns were found in animals which died/had to be euthanized due to severe clinical signs. This corresponds to a maximal viral load of the different parenchymas. These results suggest that the time frame for the immunohistochemical detection of WNV antigen might be finite and closely associated with severe clinical disease.

In conclusion both vaccines provide good partial protection when administered three times. Therefore this scheme is recommended for falcons, if feasible. Duvaxyn can be administered safely. Our data indicate that the canarypox vectored vaccine provides a better protection against viraemia and shedding, but safety concerns and adverse reactions may limit its use. In both cases viremia levels may be below the transmissibility limits for arthropods which may be considered as an additional indirect protection approach. Further studies are needed to determine the longevity of the protective effect of the vaccination and a possible cross-protection of falcons to WNV lineage 2.

## Competing interests

The authors declare that they have no competing interests.

## Authors’ contributions

The study was designed by MHG, UZ and ML with inputs also from JA and DF. JG examined the birds at the breeding centre and contributed in preparation of the experiment. JA, DF, CF, ME and UZ carried out the experiments. The results were analysed by JA, UZ and MHG and the manuscript written and corrected by JA, DF, CF, UZ, MHG and ML. All authors read and approved the final manuscript.

## Supplementary Material

Additional file 1 **Clinical scores of falcons during challenge.** Clinical score ranging from 0 to 4 is illustrated using a colour code with clinical score of 0 in white = healthy, clinical score of 1 in yellow = mildly affected, clinical score of 2 in orange = moderately affected, clinical score of 3 in red = severely affected and clinical score of 4 in dark purple = death. Grey fields indicate that the bird was taken out of the experiment at 19 or 20 days post infection (dpi) regularly. All groups presented less clinical disease than the unvaccinated control group following infection with WNV. These differences were statistically significant. Click here for file

Additional file 2 **Pathological (HE) and immunohistochemical (IHC) results for the falcons.** High IHC scores were only detected in falcons that died suddenly or had to be euthanized following infection with WNV lineage 1 NY’99. Click here for file

Additional file 3 **Results of statistical analyses.** Results of statistical analyses for the variables clinical scores, oral and cloacal shedding, viremia and viral load of brain, spleen, kidney and heart are shown. For each variable the results of each group (groups 1, 2, 3 and 4) were compared to the results of group 5. *P* values below α-levels of 0.05 were considered statistically significant. Click here for file
