# Supplementary material for: Limited efficacy of West Nile virus vaccines in large falcons (Falco spp.)
Source: Vet Res. 2014 Apr 7;45(1):41. doi: 10.1186/1297-9716-45-41 (PMC4021075; doi:10.1186/1297-9716-45-41)
Supplement: Additional file 2 — Pathological (HE) and immunohistochemical (IHC) results for the falcons. High IHC scores were only detected in falcons that died suddenly or had to be euthanized following infection with WNV lineage 1 NY’99. [file 1297-9716-45-41-S2.doc]

**Additional file 2 Limited efficacy of West Nile virus vaccines in large falcons (*Falco* spp.)**

**Pathology of control birds**

Histopathologically all falcons (7/7) had an acute mild to moderate, non-suppurative encephalitis in almost all brain areas and 4/7 falcons had also a meningoencephalitis. Scattered neuronophagia, glial nodules and/or lymphohistiocytic perivascular cuffing of varying degree were frequently found. Individual birds showed endothelial hypertrophy (3/7), perivascular accumulation of gitter cells (2/7), necrosis of single Purkinje cells (3/7) and focal malacia (1/7). Myocardial alterations (5/7) were characterized by a mild to moderate acute or subacute lymphohistiocytic necrotizing myocarditis. Splenic alterations include a nonsuppurative necrotizing arteriitis (1/7) and more non-specifically an extensive necrosis of the periarteriolar lymphoid sheats (PALS) with fibrin deposition (3/7), a severe lymphoid depletion (6/7) and a marked lymphocytolysis in the remaining follicles. Additionally 5/7 animals showed a distinct infiltration of bloated foamy macrophages in the spleen and 7/7 falcons in the liver. The liver of 6/7 birds displayed an extramedullary haematopoiesis and in 2/7 cases a mild, subacute hepatitis. Additional findings of individual falcons are mentioned in Table 1.

**Immunohistochemical results obtained by the control birds**

Three falcons (2/8 and F6) revealed WNV antigen in the brain, in severe cases most prominent in cerebrum, diencephalon and cerebellum. Heaviest WNV accumulation was found in the *Area parahippocampalis* and around ependymal spaces in cerebrum. Antigen was located intraneuronally, intraglially, intraependymally and along cell processes. The IHC examination of the heart revealed a multifocal positive staining reaction of variable degree in four (3/8 and F6) birds. WNV antigen was found intracellularly in cardiocytes, in singleton inflammatory cells and in fibrocytes of the connective tissue. A clear staining reaction was seen in singleton mononuclear cells of splenic follicles and splenic sinus of 3/8 animals. Additionally, one (1/9) bird (F51) exhibited an extensive PALS necrosis associated with a clear accumulation of viral antigen in up to 80% of the necrotic material.

## Table 1 Pathological (HE) and immunohistochemical (IHC) results for the falcons. High IHC scores were only detected in falcons that died suddenly or had to be euthanized following infection with WNV lineage 1 NY’99.

| **Group** | **Bird** | **dpi** | **Brain**  **HE/IHC** | **Heart**  **HE/IHC** | **Spleen**  **IHC** | **Inject. site**  **IHC** | **Vaccination site:**  **Quality / degree** | **Additional alterations** |
| --- | --- | --- | --- | --- | --- | --- | --- | --- |
| **Group 1**  **Inactivated**  **Boost: 4 wpv**  **Challenge: 8 wpv** | F14 | 21 | 0.5/0 | 1/0 | 0 | 0 | 0 | -- |
|  | F15 | 19 | 0/0 | 0/1 | 0 | 0 | Phlebitis/1 | Steatitis |
|  | F16 | 20 | 2/0 | 2.5/0 | 0 | 0 | 0 | -- |
|  | F17 | 9 | 0.5/1 | 3/3 | 1 | 1 | NA | Hep./Heart petechiae |
|  | F18 | 21 | 1.5/0 | 1/0 | 0 | 0 | NA | -- |
| **Group 2**  **Inactivated**  **Boost: 3+6 wpv**  **Challenge: 8 wpv** | F37 | 19 | 0.5/0 | 0/0 | 0 | 0 | Nonsupp/1 | Arter./Hep./PALS necrosis |
|  | F38 | 20 | 1.5/0 | 0.5/0 | 0 | 0 | 0 | Hep. |
|  | F39 | -- | 0/0 | 0/0 | 0 | 0 | Acute Deg./1 | Visceral gout/PALS necrosis |
|  | F40 | 19 | 0.5/0 | 0/0 | 0 | 0 | Nonsupp./1 | -- |
|  | F41 | 20 | 0/0 | 0/0 | 0 | 0 | Follicles/1 | -- |
| **Group 3**  **Recombinant**  **Boost: 4 wpv**  **Challenge: 8 wpv** | F19 | 20 | 1/0 | 1/0 | 0 | 0 | Granulomat./2 | -- |
|  | F20 | 19 | 0.5/0 | 0/0 | 0 | 0 | Granulomat./3 | -- |
|  | F21 | 21 | 2/0 | 2/0 | 0 | 0 | Granulomat./3 | Pancr. |
|  | F22 | 21 | 0/0 | 0/0 | 1 | 0 | Granulomat./2 | -- |
|  | F23 | 20 | 0/1 | 0.5/1 | 0 | 0 | Granulomat./2 | -- |
| **Group 4**  **Recombinant**  **Boost: 3+6 wpv**  **Challenge: 8 wpv** | F44 | 20 | 2/0 | 0*/1 | 0 | 0 | Granulomat./2 | Hep./Pancr./Neur |
|  | F45 | 19 | 0.5/0 | 0/0 | 0 | 0 | Granulomat./3 | -- |
|  | F46 | -- | 0/0 | 0/0 | 0 | 0 | Nonsupp./1 | Neur., Intestinal Endoparasitosis |
|  | F47 | 21 | 2/0 | 2/0 | 0 | 0 | Granulomat./3 | -- |
|  | F48 | 19 | 0.5/0 | 0/0 | 0 | 0 | Granulomat./3 | Urethritis due to Coccidiosis |
| **Group 5**  **Control**  **No vaccination** | F13 | 19 | 2/0 | 0.5/0 | 0 | 0 | ND | Neur. |
|  | F24 | 20 | 2/1 | 2/0 | 0 | 0 | ND | Arter./Hep./Neur./Pancr./Serositis |
|  | F27 | 14 | 1.5/1 | 3/0 | 0 | 1 | ND | Neph./ Ent./Neur. |
|  | F36 | 20 | 1.5/0 | 1/0 | 0 | 0 | ND | Neur. |
|  | F42 | 5 | 1/0 | 0*/0 | 1 | 2 | ND | Neur./PALS necrosis/*Petech. |
|  | F43 | 20 | 1.5/0 | 2/1 | 1 | 1 | ND | Neur./Pancr./Ent. |
|  | F51 | 8 | 0.5/0 | 0/1 | 3 | 2 | ND | Hep./Uratneph./PALS necrosis |
|  | F55 | 3 | 0/0 | 0/1 | 0 | 3 | ND | Septicaemia/PALS necrosis |
| **Pos. control/Additional bird** | F6 | 10 | 1.5/3 | 1/3 | 0 | 1 | ND | Pancr./Hep. |
| **Environmental control** | F63 | -- | 0/0 | 0/0 | 0 | 0 | NA | -- |

NA = not available; Inject. = injection; ND=not done; Nonsupp. = nonsuppurative; Granulomat. = granulomatous inflammation; *Haemorrhages; Hep. = hepatitis; Art. = arteriitis; PALS = periarteriolar lymphoid sheat; Pancr. = pancreatitis; Neur. = neuritis; Neph. = nephritis; The numbers for HE examination indicate a weak (0.5), mild (1), moderate (2) and severe (3) alteration. The score for IHC indicate no positive tissue structures (0), < 1% positive tissue structures (1), > 1% and < 5% positive tissue structures (2) and > 5% positive tissue structures. (3)
